# Spatiotemporal variation of nitrate uptake kinetics within the maize (*Zea mays* L.) root system is associated with greater nitrate uptake and interactions with architectural phenes

**DOI:** 10.1093/jxb/erw133

**Published:** 2016-04-02

**Authors:** Larry M. York, Moshe Silberbush, Jonathan P. Lynch

**Affiliations:** 1 ^1^ Department of Plant Science, The Pennsylvania State University, University Park, PA 16802, USA; 2 ^2^ Intercollege Program in Ecology, The Pennsylvania State University, University Park, PA 16802, USA; 3 ^3^ Ben-Gurion University of the Negev, J. Blaustein Institute for Desert Research/French Institute of Dryland Agricultural Biotechnology, Sede-Boqer Campus, 84990 Israel

**Keywords:** Acquisition, architecture, corn, depletion, nitrogen, plant, rhizosphere, soil.

## Abstract

Increasing maize nitrogen acquisition efficiency is a major goal for the 21st century. Nitrate uptake kinetics (NUK) are defined by *I*_max_ and *K*_m_, which denote the maximum uptake rate and the affinity of transporters, respectively. Because NUK have been studied predominantly at the molecular and whole-root system levels, little is known about the functional importance of NUK variation within root systems. A novel method was created to measure NUK of root segments that demonstrated variation in NUK among root classes (seminal, lateral, crown, and brace). *I*_max_ varied among root class, plant age, and nitrate deprivation combinations, but was most affected by plant age, which increased *I*_max_, and nitrate deprivation time, which decreased *I*_max_. *K*_m_ was greatest for crown roots. The functional–structural simulation *SimRoot* was used for sensitivity analysis of plant growth to root segment *I*_max_ and *K*_m_, as well as to test interactions of *I*_max_ with root system architectural phenes. Simulated plant growth was more sensitive to *I*_max_ than *K*_m_, and reached an asymptote near the maximum *I*_max_ observed in the empirical studies. Increasing the *I*_max_ of lateral roots had the largest effect on shoot growth. Additive effects of *I*_max_ and architectural phenes on nitrate uptake were observed. Empirically, only lateral root tips aged 20 d operated at the maximum *I*_max_, and simulations demonstrated that increasing all seminal and lateral classes to this maximum rate could increase plant growth by as much as 26%. Therefore, optimizing *I*_max_ for all maize root classes merits attention as a promising breeding goal.

## Highlight

Nitrate uptake kinetics varied among maize root classes, and simulations demonstrated that increasing the maximum uptake rate, *I*_max_, of all roots could increase plant growth by as much as 26%.

## Introduction

An increase of 100% in food production is necessary to meet the requirements of the global population of 9.7 billion predicted by 2050 (World Bank, 2014) to address global food insecurity, a defining challenge of this century (Funk and Brown, 2009). Farming more land is not a viable solution for this problem in most regions of the world (Pretty, 2008), so land use efficiency must increase dramatically. Optimization of crop nutrient acquisition efficiency is an important method with which to produce food more effectively (Lynch, 1998), especially because in much of the developing world, soil nitrogen availability is suboptimal, yet use of nitrogen fertilizer is limited (FAO, 2012). In developed nations, intensive nitrogen fertilization pollutes water and the atmosphere (Jenkinson, 2001), and in some systems only 50% of applied nitrogen is acquired by the crop (Di and Cameron, 2002). Global maize yield is greater than that of any other grain crop, and maize is grown on 177 Mha (FAO, 2012), with importance for both subsistence and commercial agriculture. Greater nitrogen acquisition efficiency in maize would improve worldwide agricultural production and mitigate environmental risks.

Nitrate is generally the most abundant form of available nitrogen in agricultural systems and acquired by crops in the greatest amounts (Miller and Cramer, 2004). The rate of nitrate absorption by a localized root segment is largely determined by nitrate uptake kinetics (NUK), which determine influx of nitrate as a function of external nitrate concentration. Epstein and Hagen (1952) first reported the use of Michaelis–Menten kinetics to describe root uptake of nutrients. Uptake kinetics were modeled as an uptake rate that saturates as the nitrate concentration increases in solution surrounding the roots with first-order unidirectional kinetics. Given these assumptions, the relationship between uptake rate and external nitrate concentration is summarized with the Michaelis–Menten parameters *I*_max,_ *K*_m_, and C_min_ (see Equation 5). *I*_max_ is the maximum influx rate of nitrate, *K*_m_ denotes the external nitrate concentration at which half of *I*_max_ is obtained, and *C*_min_ is the minimum external nitrate concentration at which net uptake may occur. The underlying mechanistic assumption of this mathematical expression is that enzymes are actively involved in the uptake process. The affinity of a transporter for its substrate is represented by *K*_m_ and determines how well the transporter operates at low substrate concentrations.

Research on NUK has occurred at three distinct levels of biological organization: transporters, root segments, and root systems. However, research integrating across these levels is rare. The most basic level is that of nitrate transporters (Quaggiotti *et al.*, 2003, 2004; Tsay *et al.*, 2007; Trevisan *et al.*, 2008), and more recently the molecular basis of nitrate uptake (Parker and Newstead, 2014). The intermediate level is at the scale of a root segment, a short section of root with many transporters in the epidermis. Transporters and their interactions with other cellular processes determine the uptake of nitrate from solution for the root segment (Lazof *et al.*, 1992; Sorgonà *et al.*, 2011). Within a root segment, even different cell types probably have unique and interacting roles for the uptake of nitrate (Gifford *et al.*, 2008). Root segments collectively form a total root system which integrates all roots to generate plant-level nitrate uptake through its interaction with soil and the shoot (Pace and McClure, 1986; Hasegawa and Ichii, 1994). Variation of NUK among root segments will determine how root segments influence total root system uptake, along with their interaction with the spatiotemporal distribution of nitrate concentration in soil, which is dynamic (Beckett and Webster, 1971). Although ammonium also contributes to plant nitrogen status, in rice NUK showed greater affinity and efficiency than ammonium uptake kinetics, and nitrate is the dominant form in most agricultural soils (Kronzucker *et al.*, 2000).

The transporters responsible for the shuttling of nitrate from external solution (soil or otherwise) into the root have been elucidated. A high-affinity (low *K*_m_) transport system (HATS) and a low-affinity (high *K*_m_) transport system (LATS) have been discovered, with transporter proteins encoded by the *NRT1* and *NRT2* gene groups, respectively, in Arabidopsis (Tsay *et al.*, 2007). In maize, *ZmNrt1* and *ZmNrt2* genes correspond to differences in uptake relating to expression levels (Quaggiotti *et al.*, 2003, 2004; Trevisan *et al.*, 2008). Recent research has supported proton-coupled transport of nitrate by *NRT1.1* and an alternating access mechanism where a central binding site reorients to expose the bound nitrate alternatively from the external to internal solution (Parker and Newstead, 2014). Furthermore, *NRT1.1* may be post-translationally modified by phosphorylation in order to change to a high-affinity state (Parker and Newstead, 2014; Sun *et al.*, 2014). HATS and LATS transporters may exist at different relative abundances in the root epidermis, and be post-translationally modified to influence kinetics, so *K*_m_ and *I*_max_ may vary independently at the root segment level.

NUK have primarily been measured using whole root systems (Pace and McClure, 1986; Hasegawa and Ichii, 1994) with little regard to possible differences among root classes, or measured on excised roots (Rao *et al.*, 1997), which introduces complications due to the wound response. In maize, differential ^15^N accumulation was demonstrated for the primary root tip, other zones of the primary root, and the primary root laterals, but neither *I*_max_ nor *K*_m_ was reported (Lazof *et al.*, 1992). In another case, *I*_max_ and *K*_m_ were determined along intact maize primary roots using a compartmented chamber, but no other classes were included (Sorgonà *et al.*, 2011). Ammonium and nitrate kinetics were determined for intact crop and tree root tips in the field by carefully removing soil and placing tips in varying solution concentrations (Bassirirad *et al.*, 1999). Determination of uptake can be based on depletion of nitrate from an external solution, or more directly based on uptake of a radiotracer such as ^13^N (Kronzucker *et al.*, 1995). To our knowledge, NUK parameters have been phenotyped across multiple maize genotypes in only one study (Pace and McClure, 1986) which determined *I*_max_ and *K*_m_ for 15 genotypes at the whole root system level. Determining how transporter properties and abundance influence NUK at the level of root segments, and how root segments interact within the whole root system to determine whole plant uptake in the context of the dynamics of soil nitrate bioavailability is necessary before natural variation in NUK can be deployed in plant breeding.

**Fig. 1.**
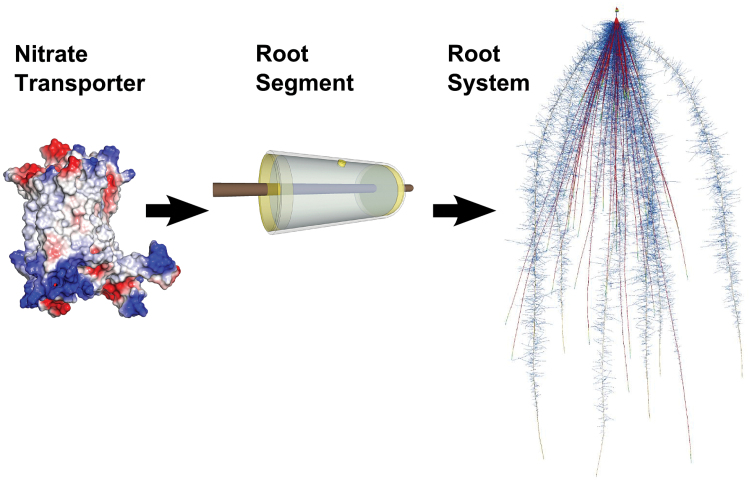
Nitrate transporters are in the epidermis of root segments which integrate to form a plant root system. NRT1.1 is shown from Parker and Newstead (2014), with permission from Macmillan Publishers Ltd: *Nature* Molecular basis of nitrate uptake by the plant nitrate transporter NRT1.1. © (2014). The root segment shows the experimental unit for measuring uptake kinetics, with a focal root segment being placed within a PVC chamber. A *SimRoot* rendering of a typical maize root system that integrates nitrate kinetics and root system architecture. The simulation is at 40 d of age, and is colored by *I*_max_ where warmer colors indicate greater *I*_max_.

Another important contributor to nitrogen acquisition efficiency is root system architecture, which is important in agricultural systems (Lynch, 1995; Ho *et al.*, 2004; Hirel *et al.*, 2007) and natural systems (Mahall and Callaway, 1992; Comas and Eissenstat, 2009) because of its effects on soil resource acquisition, plant interactions, and nutrient cycling. Throughout this manuscript, the discrete units of phenotype will be referred to as phenes (*sensu* Serebrovsky, 1925), particular values of those phenes as phene states, and conglomerations of phenes as phene aggregates (see York *et al.*, 2013). While NUK determine the potential rates of nitrate uptake by a root segment, root system architecture determines root placement in relation to soil nitrate availability, so kinetic and architectural phenes probably interact (York *et al.*, 2013) in integrated phenotypes (see Fig. 1). Understanding how root phenes influence soil resource acquisition is critical for crop improvement (Kell, 2011; Lynch and Brown, 2012). The maize root system is comprised of an embryonic root system that emerges from the seed, and whorls of nodal roots that emerge from the shoot successively as the plant grows (Hochholdinger, 2009). Many root system architectural phenes influence water and nutrient uptake and root distribution in maize, including crown root number (York *et al.*, 2013; Saengwilai *et al.*, 2014), topsoil foraging (Zhu *et al.*, 2005), crown root angle (Trachsel *et al.*, 2013), and lateral branching (Zhu and Lynch, 2004; Postma *et al.*, 2014a; Zhan and Lynch, 2015; Zhan *et al.*, 2015). Furthermore, these architectural phenes interact to increase nitrogen acquisition by maize in the field (York and Lynch, 2015), and influence competition and facilitation among plants (Zhang *et al.*, 2014).

The Barber–Cushman model (Barber and Cushman, 1981; Barber, 1984) was an early, influential computational model of nutrient acquisition by roots that uses Michaelis–Menten parameters. Barber (1984) previously described sensitivity analysis of several of the model parameters for nitrate uptake; however, the original Barber–Cushman model assumes equidistance between roots, thereby ignoring root system architecture, and assumes the soil is homogeneous with regards to nutrient concentration. However, previous work with the functional–structural model *SimRoot* indicated that the Barber–Cushman model overpredicted nitrate uptake because of an absence of nitrate leaching and no ability to simulate root competition in three dimensions (Postma and Lynch, 2011b). *SimRoot* incorporates the *SWMS_3D* model (Simunek *et al.*, 1995) for water and nitrate movement in a finite element mesh such that roots more realistically compete for nitrate.

Root plasticity is believed to be an important component of plant strategies for acclimating to soil heterogeneity and includes both morphological and physiological plasticity (Hodge, 2004). Root proliferation in nutrient-rich patches is a well-known phenomenon, though the question of ‘why do plants bother’ to proliferate in patches of highly mobile nutrients (e.g. nitrate) is still relevant (Robinson, 1996). On the other hand, physiological plasticity through the modification of uptake rates at the level of root segments and entire root systems is less well understood (Drew and Saker, 1975; Robinson, 2001). However, in general, roots respond to patches of nitrate by first increasing the uptake rate followed several days later by root proliferation (van Vuuren *et al.*, 1996). Complex transcriptomic and proteomic responses to nitrate have been observed in the maize root apex, indicating a molecular underpinning to these physiological and architectural modifications (Trevisan *et al.*, 2015). The transient nature of uptake rates followed by the permanent construction of roots is a sensible strategy to cope with nutrients that vary in time and space. Research on uptake rate plasticity has not determined *I*_max_ and *K*_m_, so the topic remains unclear.

Here is reported: (i) a novel method for measuring NUK from intact root segments within a whole root system using individual segment-specific chambers; (ii) how NUK differ among root classes and ages in a maize cultivar; and (iii) simulation results demonstrating how NUK influence plant performance and interact with root system architecture. The hypotheses were that NUK would differ among classes and would have synergistic effects with root system architecture phenes. The functional utility of spatiotemporal variation of NUK within a root system is discussed within the framework of integrating research of NUK across the levels of biological organization and implications for natural and agricultural systems.

## Materials and methods

### Empirical measurements of nitrate uptake kinetics

Maize (*Zea mays* L. Dekalb DKC44-92) seeds were germinated on germination paper soaked in 0.5mM CaSO_4_ in a dark incubator at 28 °C for 3 d. Seeds were germinated in two groups staggered 5 d apart so as to have both 15- and 20-day-old plants at the time of measurements. For 20-day-old plants, plants were deprived of nitrate, as described below, for either 2 d or 5 d before the measurements at 20 d. The seedlings were then transplanted to 30 liter hydroponics containers with 9–12 plants per container. The nutrient solution contained 1.5mM Ca(NO_3_)_2_, 0.5mM K_2_SO_4_, 0.25mM Ca(H_2_PO_4_)_2_, 0.5mM MgSO_4_, 75 µM Fe-DTPA (diethylene triamine pentaacetate), 46.25 µM H_3_BO_3,_ 9.15 µM MnCl_2_, 0.76 µM ZnSO_4_, 0.32 µM CuSO_4_, 0.51 µM H_3_MoO_4_ (Hoagland and Arnon, 1950). A few grains of Fe(NH_4_)_2_(SO_4_)_2_ salt were added weekly to prevent leaf iron deficiency symptoms. The pH was adjusted to 5.5 using KOH. The hydroponic solution was aerated using two aquarium stones attached to an air pump. The containers were placed in a greenhouse with additional light provided by a set of sodium halide bulbs to maintain 16h daylength. The remains of the pericarp and endosperm were removed 9 d after germination. The nutrient solutions were changed every week. The plants were transferred to a NO_3_-free nutrient solution, where Ca(NO_3_)_2_ was replaced by CaSO_4_, for 2 d or 5 d before measurement of NUK.

Four 15-day-old or three 20-day-old plants, depending on the experiment, were transferred to the lab in the procedure solution containing 0.5mM CaSO_4_+0.5mM K_2_SO_4_, to which 150 µM KNO_3_ was added for induction of the nitrate transport system (Hole *et al.*, 1990). This aerated solution was changed every hour for 6h. During this time, the leaves were illuminated by a 100W sodium halide bulb, which provided 103 µmol m^−2^ s^−1^ PAR. The plants were then transferred to a 40×25cm bath, which contained 2 or 3 liters of the procedure solution for 15- and 20-day-old plants, respectively, at 25 °C, with aeration. The roots were covered with a sheet of aluminum foil to avoid exposure to direct light. KNO_3_ was added to the bath to provide initial nitrate concentration between 5 µM and 150 µM on different runs.

Ten minutes later, 4cm long segments of 6.3mm (1/4 in) inner diameter polyvinyl chloride (PVC) pipe were mounted on the target root sections: 0–4cm (tip, elongation, and apical maturation zones) or 4–8cm (only the basal maturation zone) of the following root classes: seminal (from scutellar node), crown, brace, and laterals of the seminal roots (Fig 1). The crown roots sampled were from the first node, while here brace roots are defined as nodal roots from the third node that had emerged in light, so were pigmented, and were still relatively short with no lateral branching. The tubes included a small 3mm port in the middle covered with a drop of silicon sealant (Silicone II*, GE, Huntersville, NC, USA) that would later allow samplings of the inner solution with a syringe. The tube and encapsulated root were submerged in the nutrient solution which allowed solution to fill the tube completely with no air bubbles through the open ends. Then, the tubes were completely sealed on both ends with high-vacuum silicon grease in order to isolate the root segment from the solution bath. After 1h, the root on both sides of the tube was cut, the tube was removed from the bath solution, and its contents were retrieved with a syringe. The samples were stored in 6ml vials and immediately frozen. The samples were analyzed for final nitrate concentration using ion-chromatography (Dionex ICS-1100). The root sections were stored in 25% ethanol, and their length and mean diameter were determined using WinRhizo Pro software (v. 2002c, Regent Instruments, Canada).

### Michaelis–Menten calculations

Influx to the target root section may be calculated as:

(1) $$I_{n}=-\frac{V\left(C_{t}-C_{0}\right)}{A\left(t-t_{0}\right)}$$

where *I*_n_ is net influx to the root segment, *C*_0_ is the initial nitrate concentration of the bulk solution at mounting time (*t*_0_), and *C*_t_ is the nitrate concentration within the tube at sampling time (*t*); *A* is the absorbing surface area of the root segment; and *V* is the volume of the solution in the tube.

The root length that was actually exposed to the inner solution is uncertain (see Fig. 1), because the grease sealant on both sides occupies an unknown volume of the tube. The exact volume of the solution is therefore also unknown. However, the volume *V* of the solution in the tube equals the internal volume of the tube minus the volume of the grease sealing and the volume occupieds by the root. Taking *L* as the effective root length exposed to the solution, and *r* as the root radius, and assuming the root length to match that of the void:

(2) $$V=\;\pi L\left(R^{2}-r^{2}\right)$$

and

(3) A=2πrL

where *R* is the inner radius of the tube and assuming cylindrical geometry of both the tube and the root. Substituting *V* and *A* in Equation (1) with those of Equations (2) and (3) yields:

(4) $$I_{n}=-\frac{\left(C_{t}-C_{0}\right)\left(R^{2}-r^{2}\right)}{2r\left(t-t_{0}\right)}$$

Equation (4) includes the measured concentrations at the start (*t*_0_) and at the end (*t*) of the depletion trial, the radius of the tube, and that of the root. The uncertain values of the effective root length exposed to the solution and of the actual volume of the solution are not necessary, as they are expressed by measurable or provided parameters: the radius of the root may be accurately determined using WinRhizo Pro software and that of the tube is given. Using units of µmol cm^−3^ for the concentrations, cm for the radii, and s for time will result in net influx in µmol cm^−2^ s^−1^.

The influx data were plotted against mean initial nitrate concentration, from which the Michelis–Menten kinetic coefficients were calculated by non-linear curve fitting (Siddiqi *et al.* 1990):

(5) $$I_{n}=\frac{I_{max}\left(C-C_{min}\right)}{K_{m}+\left(C-C_{min}\right)}$$

where *I*_n_ is net influx to the root, *C* is concentration, and *I*_max_, *K*_m_, and *C*_min_ are parameters standing for maximal influx, concentration when *I*_n_=0.5 *I*_ma*x*_, and concentration where *I*_n_=0, respectively.

### Statistics

Michaelis–Menten parameters were fit using non-linear regression with the *nls* function in R 3.1.2 (R Core Team, 2014), which also supplied the standard error for each parameter. Confidence intervals for the models were constructed using the *predictNLS* function from the *propagate* package in R. *T*-tests were conducted for multiple comparisons of the parameters by using the standard error and number of points in the fitted model, so are provided as a best estimate of significant differences. Comparisons of fitted models for comparing across age and root classes were done using ANOVA. Simulation results are not amenable to standard statistical analyses (White *et al.*, 2014).

### Structural–functional plant modeling in SimRoot

In order to investigate the integration of NUK and root system architecture, the functional–structural plant model *SimRoot* was used (Lynch *et al.*, 1997; Postma and Lynch, 2011b). For detailed information on the structure and function of *SimRoot*, readers are referred to Postma and Lynch (2011a, b), but the most pertinent details follow. *SimRoot* simulations include both a starting seed and soil defined by soil structure, water, and nitrate properties. The seed produces root axes with properties parameterized by extensive empirical research, except for properties manipulated for the simulation experiment. In this study, all plant properties remained the same in all simulations except for NUK and architecture parameters as described below. The model includes a non-spatially explicit canopy model with expansion of leaf area leading to increased photosynthesis, and with growth rates constrained by maxima measured in real plants. Maximum growth rate is slowed proportionally as nitrogen stress increases, and nitrogen stress also increases the relative carbon allocation to the root system. The soil transport model *SWMS_3D* (Simunek *et al.*, 1995) is used to simulate water and solute movement in the soil, such that root uptake results in depletion of water and nitrate from the soil which will drive water and nitrate flux in the soil. The simulated soils include parameters affecting water and nitrate movement and include mineralization of nitrate from organic matter.

First, sensitivity analysis of the whole maize root system to *I*_max_ and *K*_m_ was conducted by varying them independently of each other, with all classes of roots having the same values of *I*_max_ and *K*_m_. *I*_max_ was varied across nine levels between 6 pmol cm^−2^ s^−1^ and 70 pmol cm^−2^ s^−1^. *K*_m_ was varied across nine levels between 5 µM and 80 µM. For both *I*_max_ and *K*_m_, the range selected includes values slightly less than and greater than the observed minima and maxima from the empirical component of this manuscript (see Table 1). In order to test the effect of variation for *I*_max_ among root classes, *I*_max_ was maintained constant at 6 pmol cm^−2^ s^−1^ for all root classes except independently increased *I*_max_ to 46 pmol cm^−2^ s^−1^, which was near the maximum observed empirically, for lateral, seminal, crown, and brace root classes. In all cases, nitrogen availability was varied between 20kg ha^−1^ and 200kg ha^−1^ across five levels, which corresponds to initial soil solution nitrate concentrations between 250 µM and 2500 µM.

**Table 1.** Michelis–Menten kinetics coefficients calculated for nitrate influx to intact roots of corn grown in hydroponics for 15 or 20 d, deprived for 2 d or 5 d prior to the determination procedure In each column, values with the same letter are not significantly different at *P*≤0.05 levels according to the paired *t*-test. Combinations where net influx (In) responds linearly to the concentration (*C*) are represented by the linear regression.

Age (d)	Deprivation (d)	Root class	Position^*a*^ (cm)	*I*_max_(pmol cm^−2^ s^−1^)	*K*_m_(µM)	*C*_min_(µM)	*R* ^2^	*n*
15	2	Lateral	0–4	14.66 g	2.68 d	1.70 c	0.82	10
20	2	Lateral	0–4	45.25 a	10.67 c	4.40 ab	0.68	9
			4–8	35.81 ab	17.25 bc	1.64 c	0.84	5
20	5	Lateral	0–4	30.54 ab	21.33 bc	4.69 ab	0.55	15
			4–8	In=0.4044×C+2.4268			0.79	8
15	2	Seminal	0–4	26.64 bc	10.50 c	2.82 bc	0.79	14
			4–8	22.17 c	16.10 bc	2.06 c	0.64	14
20	2	Seminal	0–4	33.76 b	10.31 cd	3.79 a	0.70	18
			4–8	30.03 ab	6.72 cd	4.68 ab	0.67	9
15	2	Crown	0–4	14.02 d	15.70 bc	2.36 c	0.82	14
			4–8	24.30 c	52.21 a	0.98 c	0.90	14
20	2	Crown	0–4	41.25 a	32.74 ab	6.20 a	0.82	9
			4–8	46.52 a	45.49 ab	4.28 ab	0.95	18
20	5	Crown	0–4	In=0.2502×C+1.9036			0.66	18
			4–8	In=0.3365×C+0.2930			0.95	18
20	2	Brace	0–4	16.47 cd	28.21 ab	3.22 abc	0.81	6
			4–8	In=0.3357×C+0.9501			0.92	7
20	5	Brace	0–4	In=0.2183×C–0.4755			0.73	13
			4–8	In=0.2949×C+1.2704			0.86	5

^*a*^ Distance from the root tip.

Architectural phene states that increase root length density would be expected to increase the overlap in nitrate depletion zones which are also made larger by increases in *I*_max_, thereby decreasing any benefit *I*_max_ would have in the absence of increased inter-root competition. All levels of *I*_max_ were factorially combined with four levels of nodal root number (between eight and 46), four levels of nodal root angle (between 20 ° and 80 ° from horizontal), and five levels of lateral root branching (between 2 and 20 laterals cm^−1^), which represent the ranges observed in the field for these phenes (Trachsel *et al.*, 2011). In all cases, nitrate availability was varied between 20kg ha^−1^ and 200kg ha^−1^ across five levels. The importance of *I*_max_ during interplant competition was evaluated by simulating two plants either with the same *I*_max_ (intraphenotypic competition) or with different *I*_max_ (interphenotypic competition), with the two levels of *I*_max_ being 46 pmol cm^−2^ s^−1^ and 6 pmol cm^−2^ s^−1^, which represent the maximum and minimum values, respectively, observed in the empirical experiments. All simulations had two replicates, and standard error was <1% of the mean in all cases because *SimRoot* is fundamentally a deterministic model, with variation only caused by small random changes to growth angles at each time step.

## Results

### Empirical

**Fig. 2.**
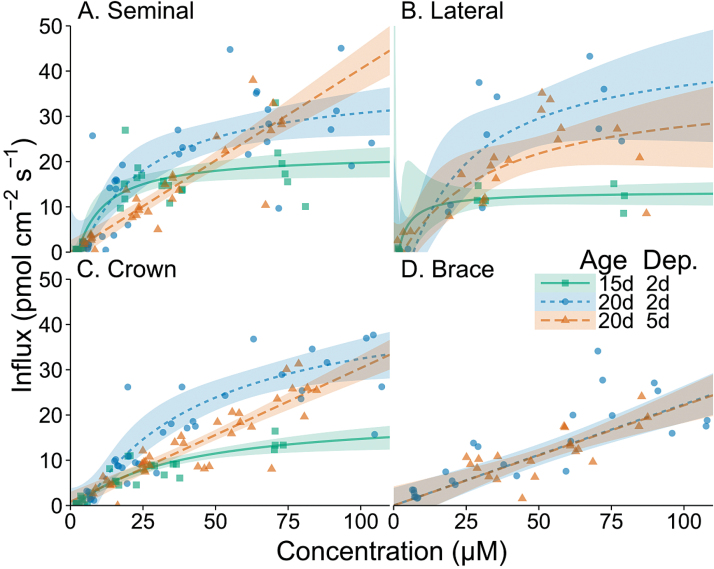
Nitrate influx at varying concentrations of nitrate in seminal, lateral, crown, and brace root classes of maize. Nitrate influx is compared between 15-day-old (15d) and 20-day-old (20d) plants deprived of nitrate for 2 d before measurements, and between 20-day-old plants at either 2 d of nitrate deprivation (2d) or 5 d of nitrate deprivation (5d). Points represent individual observations, lines represent fitted Michaelis–Menten models, and bands represent 90% confidence intervals.

**Fig. 3.**
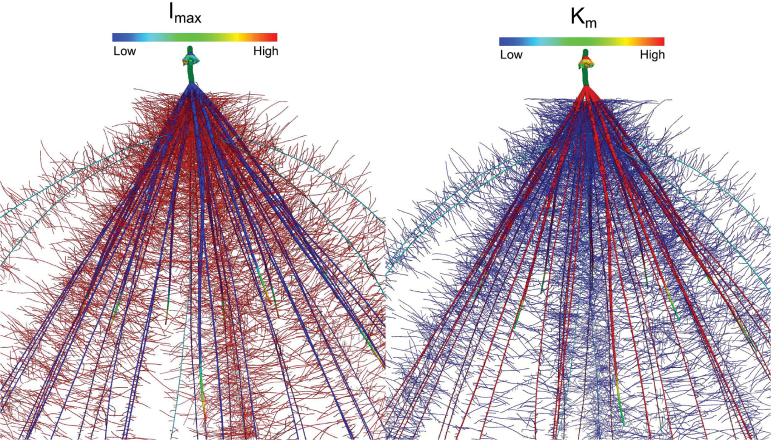
An example of variation of *I*_max_ (A) and *K*_m_ (B) within the maize root system is depicted using *SimRoot*. Variation is shown among root classes and positions as based upon the empirical data.

In order to quantify nitrate uptake kinetics among maize root classes, 4cm long PVC tubes were fitted around root segments, sealed on both ends, and solutions with varying concentrations of nitrate were added. After 1h, the difference in nitrate concentration was assumed to be net nitrate uptake, and from these data Michaelis–Menten parameters were fitted. Nitrate influx was influenced by external nitrate concentrations and root classes, exhibiting both Michaelis–Menten and linear relationships (Fig. 2). *I*_max_ varied among root classes, root position, plant ages, and number of days of nitrate deprivation (Table 1; Fig. 3), with the slowest *I*_max_ being 14.02 pmol cm^−2^ s^−1^ observed in the 0–4cm region of crown roots at 15 d of age after 2 d of nitrate deprivation, and the greatest *I*_max_ being 46.52 pmol cm^−2^ s^−1^ observed for crowns in the 4–8cm region at 20 d of age after 2 d of nitrate deprivation. On average, there were no significant differences in *I*_max_ among root classes, although differences exist at some positions, age, and deprivation levels (Table 1). In general, position along a root axis did not have a large or a consistent effect on *I*_max_. *I*_max_ increased 93% from 20.36 pmol cm^−2^ s^−1^ to 39.36 pmol cm^−2^ s^−1^ from 15- to 20-day-old plants, respectively (*P*=0.002). The only general trend for *K*_m_ was being consistently lower for seminal and lateral roots compared with crown roots (*P*=0.003), with an average of 11.9 µM for seminal and lateral roots and an average of 36.5 µM for crown roots. In five of six cases, 5 d of nitrate deprivation led to slow uptake relative to 2 d of deprivation and a linear relationship between external nitrate concentration and uptake.

### Simulation

**Fig. 4.**
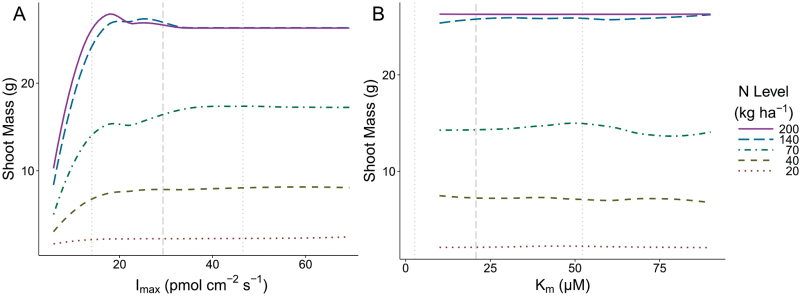
In order to conduct *I*_max_ sensitivity analysis (A) and *K*_m_ sensitivity analysis (B) on shoot growth, maize plants were simulated with a range of nine parameter values growing in soils at five nitrogen levels using *SimRoot*. The line type indicates the nitrogen level in which the simulations grew and are smoothed with loess for ease of interpretation. Vertical dashed lines indicate the minimum, average, and maximum values from the empirical study.

The empirical data described above were used to parameterize *SimRoot* to compare the effects of varying NUK on uptake and the interactions of kinetics with root system architecture. Sensitivity analysis for *I*_max_ (Fig. 4A) showed that increasing *I*_max_ increased shoot mass, but generally shoot mass reached an asymptote by 40 pmol cm^−2^ s^−1^, which was near the maximum value observed empirically. In the lowest level of nitrogen, shoot dry mass increased 54% from the lowest to highest value of *I*_max_, while at the highest level of nitrogen, there was a 183% increase. The response to increasing *I*_max_ is made more complex by the simulated plant’s response to stress, such that the shoot mass response to increasing *I*_max_ fluctuates. Sensitivity analysis for *K*_m_ (Fig. 4B) demonstrated less effect on plant performance across all nitrogen levels than did *I*_max_, with only an 8% increase in shoot dry weight at the lowest level of nitrogen (20kg N ha^−1^), comparing the greatest value of *K*_m_ with the least. At the second most severe level of nitrogen stress (40kg N ha^−1^), there was a 12% increase in shoot dry weight associated with decreasing *K*_m_.

**Fig. 5.**
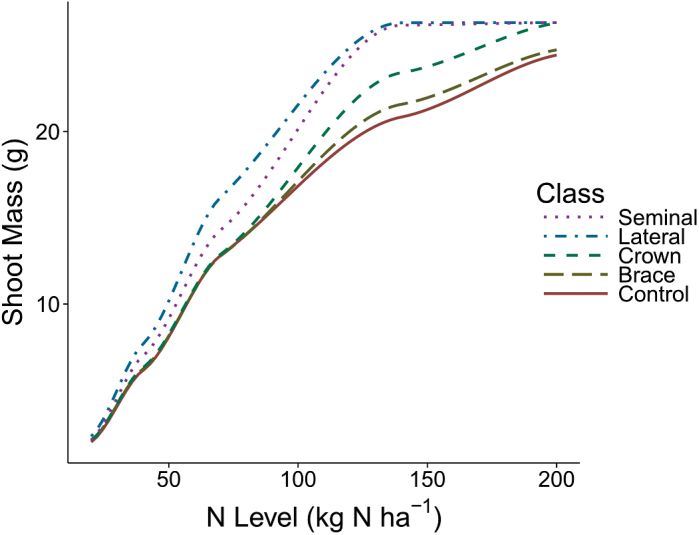
In order to test the dependency of shoot growth on the *I*_max_ of specific root classes, maize plants were simulated using *SimRoot* with variation in the *I*_max_ of different root classes across five levels of nitrogen. *I*_max_ was held constant for all root classes at 6 pmol cm^−2^ s^−1^, which was near the minimum observed, except that a focal root class was independently increased to 46 pmol cm^−2^ s^−1^, which is near the maximum observed. The line type indicates the focal root class that had increased *I*_max_ and are smoothed with loess for ease of interpretation. The control simulations have all root classes set to the slower *I*_max_.

**Fig. 6.**
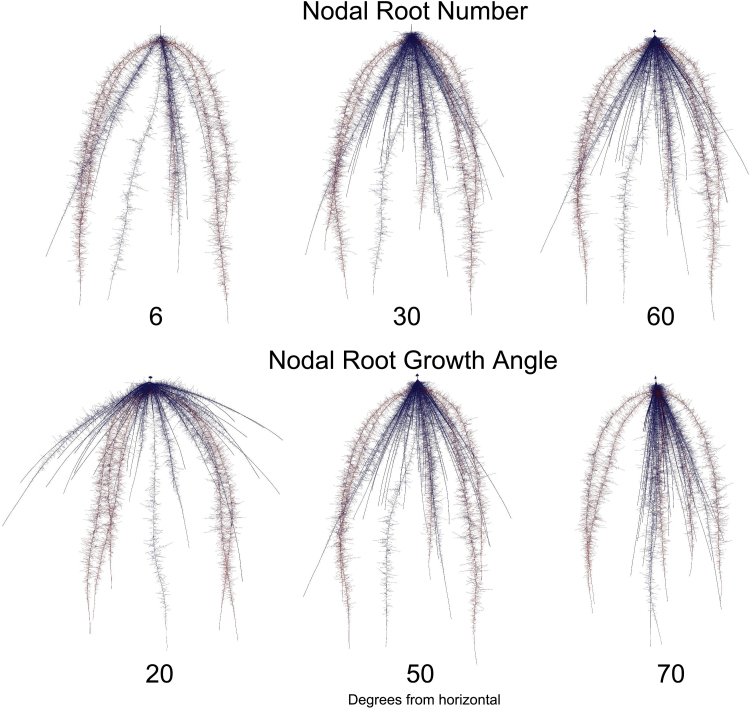
The interactions of *I*_max_ with metabolic-influential and metabolic-neutral phenes were tested using *SimRoot*. Here, example variation in nodal root number and nodal root growth angle is depicted with simulated maize root systems as examples of influential and neutral phenes, respectively. Nodal roots are shown in blue, with the primary and seminal roots in red. See Fig. 7 for simulation results.

**Fig. 7.**
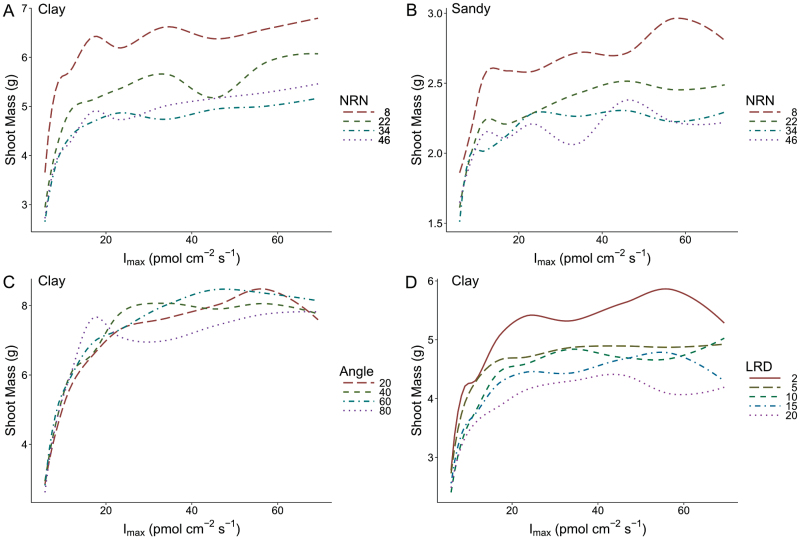
In order to test the interaction of *I*_max_ and nodal root number (NRN, A), nodal root growth angle (angle, C), and lateral root density (LRD, D), simulations of maize were conducted varying *I*_max_ across nine values with root systems with varying levels of the respective architectural phene (line type) at low levels of nitrogen (20kg N ha^−1^). In order to look at the influence of soil, further simulations were conducted for the interaction of *I*_max_ with NRN in a sandy soil at the same low nitrogen level (B).

The *I*_max_ dependency for a specific root class (Fig. 5) was demonstrated by holding all other root classes to a slow *I*_max_, 6 pmol cm^−2^ s^−1^, while increasing the *I*_max_ of the focal root class to the greatest empirically observed *I*_max_, 46 pmol cm^−2^ s^−1^. Shoot dry weight was most dependent on lateral root *I*_max_, followed by seminal, crown, and brace root classes. Across all levels of nitrogen, increasing the *I*_max_ of all lateral and seminal classes increased plant growth between 7% and 26%, with the greatest gains at moderate levels of nitrogen fertlization. The utility of *I*_max_ for shoot growth will depend on the phenotypic background in which it exists, so its interactions were modeled with three root system architectural phenes: nodal root number (Figs 6, 7A), nodal root angle (Figs 6, 7C), and lateral root branching (Fig. 7D). In general, there was relatively little interaction between *I*_max_ and the architectural phenes, such that increasing *I*_max_ generally increased shoot growth regardless of the root system architectural background in which it was expressed. On average, the range of shoot growth influenced by *I*_max_ was greater than the range of shoot growth as influenced by root system architecture.

**Fig. 8.**
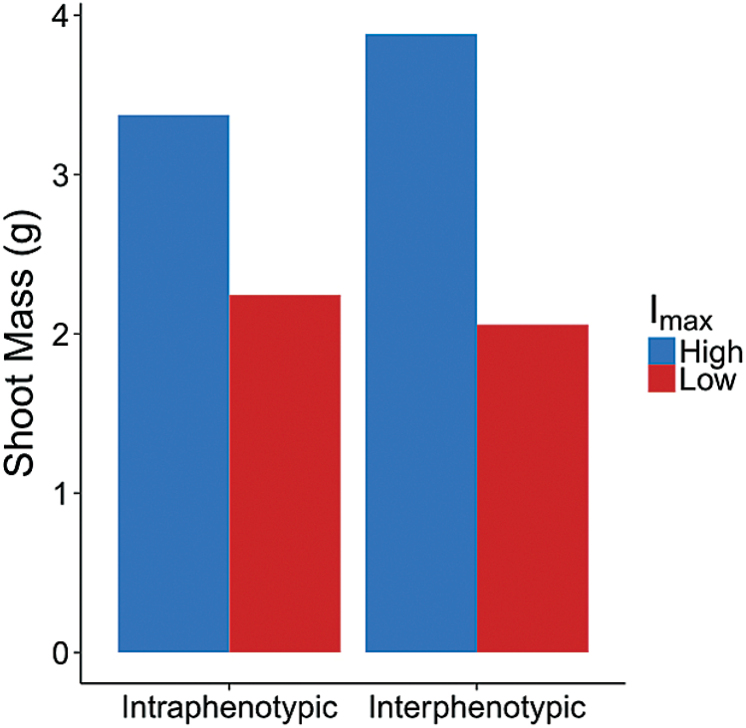
The results of competition between plants with the same *I*_max_ (intraphenotypic) or different *I*_max_ (interphenotypic). The high *I*_max_ was 46 pmol cm^−2^ s^−1^ and low *I*_max_ was 6 pmol cm^−2^ s^−1^, which represent the maximum and minimum values observed in the empirical experiments.

At the lowest level of nitrogen (20kg N ha^−1^), plants had less shoot mass in the sandy soil with high leaching than in the clay soil (Fig. 7B). Soil type did not influence the general trend of increasing *I*_max_ benefitting plant growth, but growth in sandy soil did tend to shift the local optima to greater values of *I*_max_. Under conditions of interphenotypic competition with plants with high and low *I*_max_ grown together, high *I*_max_ plants had 15% more shoot mass, while low *I*_max_ plants grew 9% less shoot mass relative to their shoot masses during intraphenotypic competition (Fig. 8).

## Discussion

NUK varied among root classes, with *I*_max_ being greatest for lateral and crown roots and *K*_m_ being least for lateral and seminal roots. Variation for NUK among root classes has not previously been documented for several root classes and ages. Older plants had greater *I*_max_ and similar *K*_m_ regardless of root class. Plants deprived of nitrate before uptake measurements had decreased *I*_max_, and a linear response to nitrate rather than a saturating response to nitrate. Indeed, because plants grown in lower concentrations of nitrate have a low *I*_max_, induction of the nitrate uptake mechanisms by placing plants in greater concentrations of nitrate is often used in NUK experiments (discussed in Kronzucker *et al.*, 1995). These results showing that plant nitrogen demand relates to NUK are consistent with other reports (Garnett *et al.*, 2013). The linear response of the nitrate-deprived plants may relate to the plant having a greater reliance on the LATS, which is known to have a linear response (Glass *et al.*, 1992; Touraine and Glass, 1997) possibly because of passive uptake in a channel-like state when the cytoplasmic nitrate concentration is low (Wang *et al.*, 2012), which may be especially true in the case of more nitrate-deprived plants. Lateral roots had greater *I*_max_ than their parent roots, possibly because lateral roots dominate total root system length and are responsible for the majority of nutrient uptake, as confirmed in the simulation component. The differences among root classes and plant ages demonstrate that spatiotemporal variation of NUK within the root system is an important phenomenon in need of further characterization.

In the simulations, *K*_m_ had relatively less effect on shoot mass than *I*_max_, but increases in shoot mass of 10% in stressful soils at 40 d of growth with decreased *K*_m_ represent a potential opportunity, especially because this increased growth will compound over time. Increasing *I*_max_ was associated with more than a doubling of shoot mass in some simulations. Increasing *I*_max_ had a complex effect on shoot mass at lower N levels because greater values of *I*_max_ allowed nitrate to be acquired in sufficient amounts, which decreased plant stress during early plant growth. *SimRoot* increases the relative allocation of carbon to the root system compared with the shoot when the plant experiences nitrogen stress, and decreases the relative allocation to the root system when stress is alleviated (Postma and Lynch, 2011a). However, this stress response may not always optimize plant growth because root growth is irreversible, and compensating with new growth is a slow process (Postma *et al.*, 2014b). A greater *I*_max_ value allows a plant to acquire adequate nitrate during early growth so relatively less mass will be allocated to the root system. As the shoot grows and demands more nitrogen, the smaller root system cannot meet this demand even at the greater *I*_max_ value, so the plant becomes stressed again and photosynthesis cannot maintain shoot growth. However, in many cases, if *I*_max_ is increased further, this stress can be alleviated by the increased N uptake per root length. However, all simulations end at 40 d, so plants are at different levels of nitrogen stress and compensation through root growth. This behavior is difficult to predict and exaggerated when interacting with phenes that influence carbon economy, such as nodal root number and lateral root branching when compared with a carbon-neutral phene like nodal root angle, which had a smoother response. In the simulation model ROOTMAP, the plasticity of NUK was found to contribute greatly to the uptake of herringbone (sparsely branched) type root systems, but with little contribution to total nitrate uptake of dichotomous (greatly branched) type root systems in simulations where nitrate supply was heterogeneous (Dunbabin *et al.*, 2004). In general, greater *I*_max_ should have more benefit when combined with phene states that decrease overall root system density, such as decreased nodal root number, decreased lateral branching, and moderate rooting angles. However, this prediction requires the assumption that increasing *I*_max_ will increase the size of nitrate depletion zones, which needs to be tested empirically.

In the original sensitivity analysis for nitrate uptake from the Barber–Cushman model, nitrate uptake was particularly sensitive to the growth rate of roots, *I*_max_, and the root radius (Barber, 1984). The model was scarcely influenced by the mean root distance (root density) or the initial concentration of nitrate. The model was completely insensitive to *K*_m_. Barber’s sensitivity analysis had a high initial nitrate concentration which explains the linear response of nitrate uptake to increasing *I*_max_, and this relationship did not reach an asymptote as in the current *SimRoot* model. The *I*_max_ used in Barber’s analysis was derived from whole root system uptake in maize in a silt loam soil, and was equivalent to 10 pmol cm^−2^ s^−1^, so even when doubled as part of Barber’s sensitivity analysis the asymptotic point of ~40 pmol cm^−2^ s^−1^ was not reached. The *K*_m_ used by Barber was 25 µM, in the mid-range of that used here, so the complete insensitivity in the Barber model was because of the high nitrate concentrations and short duration, whereas in the *SimRoot* simulations at low nitrogen or after uptake of most of the available nitrate, *K*_m_ can have a small effect on nitrate uptake. The current simulations demonstrate the importance of nitrate kinetic parameters for specific root segments and additive effects with root system architecture.

The current experiments and simulations demonstrate that the spatial heterogeneity of both root and soil processes is important. Measurements of NUK have most often been performed on whole root systems, which aggregates the properties of more and less active roots. However, because laterals have greater *I*_max_ and interact with root system architecture, variation within root systems must be considered. Waisel and Eshel (2002) stressed the importance of measuring variation among the ‘smallest distinguishable units’ for many physiological aspects of the root system in order to understand fully the functioning of entire root systems. Likewise, the relationship between NUK measured in nutrient solution and actual values in soil is unknown. However, the present simulations demonstrate that soil type does impact plant growth and its relationship to NUK. Fuller consideration of the complexity of both root systems and the soil is necessary for applying NUK to plant breeding or in understanding natural ecosystems.

This empirical work did not consider plasticity of NUK in nutrient patches, nor was such plasticity included in the model. Previous studies of nitrate uptake plasticity only measured rates of uptake in uniform soil or in a patch; these studies did not measure the Michaelis–Menten parameters (Drew and Saker, 1975; van Vuuren *et al.*, 1996; Fransen *et al.*, 1999). Our experimental procedure demonstrates that measuring *I*_max_ and *K*_m_ is possible for localized root segments other than root tips, so this method has broad applicability in the study of uptake plasticity. Indeed, parameterization of our model to include plasticity of *I*_max_ and *K*_m_ is impossible because these parameters have not been measured in response to nitrate patches. Future research on nitrate uptake plasticity must measure NUK in more detail, not only total nitrogen accumulated divided by root length or mass. In the absence of any metabolic costs, *I*_max_ and *K*_m_ would be maximized and minimized, respectively, at all times and in all soil domains. However, that the influx rate is often increased in high nitrate patches in otherwise low nitrate soil implies that there is a cost to maintaining the molecular apparatus or tissue developmental status for maximal nitrate uptake rates. The costs of NUK must be considered in greater detail. In a cost–benefit analysis, *I*_max_ should be increased until the cost of increasing *I*_max_ another increment exceeds the benefit of acquired nitrate (Bloom *et al.*, 1985; Lynch and Ho, 2005). With a constant *K*_m_ (which might generally be accurate), increasing *I*_max_ has greater effects when external nitrate concentration is high. Thus, given that there is a cost to increasing *I*_max_, *I*_max_ can be predicted to be lower in soil with low concentrations of nitrate, and greater in soil with higher concentrations of nitrate. The situation is made even more complex when considering that root system architectural phenes exhibit plasticity as well, and will probably interact through plant metabolism (York *et al.*, 2013). The plasticity of NUK deserves more attention as a focus of research.

This study focused on variation of NUK among root classes and ages, and how this variation affects total root system uptake. The demonstration of spatiotemporal variation in kinetics implies developmental and genetic control through unknown processes that must affect the relative abundances of different types of transporters and other processes affecting nitrate uptake, as discussed below. The use of transgenic mutants with transporter gene insertions and knockout mutants would not be appropriate for documenting and understanding natural variation of intraroot system NUK and its functional utility because such mutants typically have a limited range of functional states and are mostly useful for confirming the role of a gene in a functional process. Since its discovery in 2012, the CRISPR/Cas9 system that allows targeted genome editing has been implemented in Arabidopsis, tobacco, sorghum, rice, and wheat (Lozano-Juste and Cutler, 2014). CRISPR/Cas9 technology can be used to generate unavailable mutant lines, or even entire genome-wide knockout libraries *de novo* (Shalem *et al.*, 2014). This research will benefit from the screening of multiple genotypes for these phenes. Root segment NUK are expected to have complex, quantitative control because they are an aggregate created through the integration of many other phenes, as discussed below.

Functional–structural plant modeling is an invaluable tool for the study of the functional utility of root system phenes (Dupuy *et al.*, 2010), including root NUK and interactions with other root phenes. Root system simulation models that include nutrient uptake such as *SimRoot*, *ROOTMAP*, *SPACSYS*, *R-SWMS*, and *RootBox* (reviewed in Dunbabin *et al.*, 2013) will be of great utility in the study of the functional ramifications of changes in nitrate *I*_max_ and *K*_m_. Simulations allow the exploration of NUK and their interactions with other plant phenes in more combinations of climates, soil types, and nutrient levels than is possible in greenhouse and field studies, due to labor and financial constraints. Genetic and physiological constraints may make it difficult or impossible to study some phene state combinations empirically, but they can still be modeled. In an iterative fashion, simulations allow researchers to focus their empirical studies on the most fruitful phenes and phene interactions, while the information gained from empirical studies refines the models (Wullschleger *et al.*, 1994). The lack of strong interactions between NUK and root architectural phenes in this study may be affected by a lack of nitrate uptake metabolic costs, such as protein synthesis and osmotic regulation, which is a knowledge gap discussed more below. Including these costs in simulation models will be an important contribution to understanding utility of NUK for total root system nitrate uptake.

Understanding NUK must occur within the broader context of ecological interactions. Physiological plasticity of NUK may be a method for plants to respond quickly to patches or pulses of nitrate before roots are able to proliferate through branching and growth (reviewed by Hodge, 2004). During competition, plants with greater *I*_max_ may acquire more nitrate than their competitor, as demonstrated in this study’s simulation component. Despite construction costs of transporters and energetic costs associated with nitrate uptake, acquiring resources before a competitor may increase relative fitness and answer the question of ‘why plants bother’ to proliferate roots and increase NUK (Hodge *et al.*, 1999). In another simulation study, NUK ranked highly among many root and soil properties for their influence on crop–weed competition (Dunbabin, 2007). Increasing fitness relative to competitors is important in natural systems, but can lead to a ‘tragedy of the commons’, a prediction of game theory where plants overproliferate roots relative to the optimal amount of roots to maximize uptake efficiency (Gersani *et al.*, 2001). In contrast, avoidance of this overproliferation might be important for agriculture systems where optimizing yield rather than fitness of the focal crop is the goal (Zhang, 1999). Similarly, considering the costs of transporter construction and uptake energetics, there may be greater transporter redundancy and uptake costs when optimizing relative fitness in natural systems than in agricultural systems where nutrient uptake efficiency may be more important.

*I*_max_ has been known to be an important factor influencing nitrate uptake for 50 years (Lycklama, 1963; Rao and Rains, 1976; Siddiqi *et al.*, 1990). However, *I*_max_ has never been a target of a public plant breeding program, and significant knowledge gaps remain in understanding the functioning of *I*_max_ across biological levels of organization. Root segment *I*_max_ is a phene aggregate influenced by more fundamental processes. Understanding nitrate uptake necessitates formalizing the relationship between the uptake observed for a root segment on per length, area, or weight basis, and the kinetics observed for the respective individual transporters. NUK values of root segments are necessarily phene aggregates influenced by the number and types of nitrate transporters in the epidermis of a root segment, and the developmental state of that root segment in terms of the viability of the epidermis and cortex. Although the relationship between root segment *I*_max_ for nitrate and the number of nitrate transporters is not known, a linear 1:1 relationship between transporter surface density and overall uptake was found for another transporter (Garcia-Celma *et al.*, 2013). Root segment *I*_max_ may be related to the combined *I*_max_ of all individual transporters of various identities. Recently, expression of *NRT2* from Chrysanthemum in Arabidopsis resulted in the doubling of nitrate uptake in nutrient solution, while additionally expressing *NAR2* from Chrysanthemum resulted in a quadrupling of nitrate uptake in nutrient solution (Gu *et al.*, 2014). The number of nitrate transporters is related to transcription levels as well as post-transcriptional and post-translational processes (Wirth *et al.*, 2007; Gu *et al.*, 2014) so understanding the regulation of transporter generation is imperative for understanding how NUK are determined at the root segment level. Root segment *K*_m_ must be influenced by the relative abundance of HATS and LATS transporters, possibly the weighted average of constituent transporter *K*_m_ values based on abundance. More research is needed to clarify how the absolute number of the various nitrate transporters and their relative proportions are regulated by gene redundancy, transcription levels, and post-transcriptional and post-translational processes.

Ultimately, however, *I*_max_ and *K*_m_ of transporters occur at the molecular level, and what specific properties of the transporters are responsible for variation in transporter *I*_max_ and *K*_m_ are not known. Variation in transporter kinetics may exist as influenced by gene variants, or alleles, about which little is known. Parker and Newstead (2014) suggest that phosphorylation of a specific residue within *NRT1.1* allows greater flexibility of the enzyme’s mobile site and, so, greater nitrate uptake. However, in that case, the same phosphorylation event may interfere with the nitrate-binding site and increase the *K*_m_ (Parker and Newstead, 2014). If so, there is reasonable evidence that modifying or selection of transporters may be possible for greater uptake rates and binding affinities, or that natural variation in uptake parameters might exist at the transporter level. The energetics of the secondary active transport process for nitrate uptake must also be considered: given the stoichiometry of the plasma membrane H^+^ ATPase proton pumping (Sze *et al.*, 1999) and nitrate transporter uptake (Parker and Newstead, 2014), every nitrate ion absorbed requires at least 1 ATP molecule to maintain the proton gradient. Veen (1980) determined that the respiration required for nitrate uptake accounted for 20% of total plant respiration in maize. In barley, Bloom *et al.* (1992) demonstrated that 5% of root respiration is devoted to nitrate absorption and 15% to assimilation. The construction cost of transporters may be estimated based on their abundance and turnover, as well as the respiration required for their synthesis and shuttling to the epidermis external membrane. As described above, understanding the construction and maintenance costs of transporters along with the costs of uptake energetics is necessary to inform simulation modeling for optimizing NUK in whole root systems, and to understand competitive dynamics in natural and agricultural systems.

### Conclusion

Several approaches are needed in order to use NUK phenes in breeding programs. High-throughput phenotyping approaches may be used for measurements of this phene aggregate at the root segment level method used in the current study. Phenotyping of root segment NUK coupled to genome-wide association studies could prove to be a very powerful approach to discover quickly genomic regions associated with optimal kinetics and to use those in breeding programs. Since lateral roots have the greatest uptake rate and comprise the majority of the maize root system, lateral roots would be a sensible target of root segment NUK phenotyping. Increasing nitrate uptake efficiency and optimizing kinetics based on knowledge of nitrate transporters have long been proposed as a method to transform agriculture. In the simulations, plant growth was more influenced by *I*_max_ than by *K*_m_ in realistic virtual soils, so *I*_max_ may be a more important focus of future research. The optimal *I*_max_ will be defined as the point where the marginal benefit equals the marginal cost (Bloom *et al.*, 1985), and both benefits and costs associated with increasing *I*_max_ have significant knowledge gaps. The empirical results indicated that for lateral roots, only the root tips of 20-day-old plants operate at the greatest *I*_max_ level observed, which is a small percentage of all lateral root length and ages. Seminal roots generally operated at about half the maximum observed *I*_max_. The modeling results suggest that across all levels of nitrogen fertilization, lateral and seminal roots independently contributed between 7% and 26% gains in shoot mass. Targeting these root classes for greater *I*_max_ at all ages and positions could lead to substantial improvements in yield. Leveraging high-throughput phenotyping, simulation modeling, genomic analysis, and laboratory molecular research together will allow agricultural scientists to realize the promise of increasing nitrate acquisition efficiency and provide one component of the solution to the challenge of global food insecurity.

## References

[CIT0001] Barber SA 1984 Soil nutrient bioavailability . Chichester: John Wiley & Sons.

[CIT0002] Barber SA Cushman JH 1981 Nitrogen uptake model for agronomic crops. Modeling wastewater renovation: land treatment . New York: Wiley Interscience, 382–489.

[CIT0003] Bassirirad H Prior SA Norby RJ Rogers HH 1999 A field method of determining NH4 and NO3 uptake kinetics in intact roots: effects of CO2 enrichment on trees and crop species. Plant and Soil 217, 195–204.

[CIT0004] Beckett PHT Webster R 1971 Soil variability: a review. Soils and Fertilizers 34, 1–15.

[CIT0005] Bloom AJ Chapin III FS Mooney HA 1985 Resource limitation in plants—an economic analogy. Annual Review of Ecology and Systematics 16, 363–392.

[CIT0006] Bloom AJ Sukrapanna SS Warner RL 1992 Root respiration associated with ammonium and nitrate absorption and assimilation by barley. Plant Physiology 99, 1294–1301. 16669035 10.1104/pp.99.4.1294 PMC1080623

[CIT0007] Comas LH Eissenstat DM 2009 Patterns in root trait variation among 25 co-existing North American forest species. New Phytologist 182, 919–928. 19383105 10.1111/j.1469-8137.2009.02799.x

[CIT0008] Di HJ Cameron KC 2002 Nitrate leaching in temperate agroecosystems: sources, factors and mitigating strategies. Nutrient Cycling in Agroecosystems 46, 237–256.

[CIT0009] Drew MC Saker LR 1975 Nutrient supply and the growth of the seminal root system in barley. II. Localized, compensatory increases in lateral root growth and rates of nitrate uptake when nitrate supply is restricuted to only part of the root system. Journal of Experimental Botany 26, 79–90.

[CIT0010] Dunbabin V 2007 Simulating the role of rooting traits in crop–weed competition. Field Crops Research 104, 44–51.

[CIT0011] Dunbabin VM Postma JA Schnepf A Pagès L Javaux M Wu L Leitner D Chen YL Rengel Z Diggle AJ 2013 Modelling root–soil interactions using three-dimensional models of root growth, architecture and function. Plant and Soil 372, 93–124.

[CIT0012] Dunbabin V Rengel Z Diggle AJ 2004 Simulating form and function of root systems: efficiency of nitrate uptake is dependent on root system architecture and spatial and temoral variability of nitrate supply. Functional Ecology 18, 204–211.

[CIT0013] Dupuy L Gregory PJ Bengough AG 2010 Root growth models: towards a new generation of continuous approaches. Journal of Eperimental Botany 61, 2131–2143. 10.1093/jxb/erp389 20106912

[CIT0014] Epstein E Hagen CE 1952 A kinetic study of the absorption of alkali cations by barley roots. Plant Physiology 27, 457–474. 16654471 10.1104/pp.27.3.457 PMC547952

[CIT0015] FAO (Food and Agriculture Organization). 2012 FAOSTAT Online Database, http://faostat3.fao.org/.

[CIT0016] Fransen B Blijjenberg J De Kroon H 1999 Root morphological and physiological plasticity of perennial grass species and the exploitation of spatial and temporal heterogeneous nutrient patches. Plant and Soil 211, 179–189.

[CIT0017] Funk CC Brown ME 2009 Declining global per capita agricultural production and warming oceans threaten food security. Food Security 1, 271–289.

[CIT0018] Garcia-Celma J Szydelko A Dutzler R 2013 Functional characterization of a ClC transporter by solid-supported membrane electrophysiology. Journal of General Physiology 141, 479–491. 23478993 10.1085/jgp.201210927 PMC3607819

[CIT0019] Garnett T Conn V Plett D 2013 The response of the maize nitrate transport system to nitrogen demand and supply across the lifecycle. New Phytologist 198, 82–94. 23398565 10.1111/nph.12166

[CIT0020] Gersani M Brown JS Brien EEO Maina GM Abramsky Z O’Brien EE 2001 Tragedy of the commons as a result of root competition. Journal of Ecology 89, 660–669.

[CIT0021] Gifford ML Dean A Gutierrez RA Coruzzi GM Birnbaum KD 2008 Cell-specific nitrogen responses mediate developmental plasticity. Proceedings of the National Academy of Sciences, USA 105, 803–808. 10.1073/pnas.0709559105 PMC2206617 18180456

[CIT0022] Glass ADM Shaff JE Kochian L V 1992 Studies of the uptake of nitrate in barley: IV. Electrophysiology. Plant Physiology 99, 456–463. 16668907 10.1104/pp.99.2.456 PMC1080484

[CIT0023] Gu C Zhang X Jiang J Guan Z Zhao S Fang W Liao Y Chen S Chen F 2014 Chrysanthemum CmNAR2 interacts with CmNRT2 in the control of nitrate uptake. Scientific Reports 4, 1–8. 10.1038/srep05833 PMC5376060 25060485

[CIT0024] Hasegawa H Ichii M 1994 Variation in Michaelis–Menten kinetic parameters for nitrate uptake by the young seedlings in rice (Oryza sativa L.). Breeding Science 44, 383–386.

[CIT0025] Hirel B Gouis J Le Ney B Gallais A Le Gouis J 2007 The challenge of improving nitrogen use efficiency in crop plants: towards a more central role for genetic variability and quantitative genetics within integrated approaches. Journal of Experimental Botany 58, 2369–2387. 17556767 10.1093/jxb/erm097

[CIT0026] Ho MD Mccannon BC Lynch JP 2004 Optimization modeling of plant root architecture for water and phosphorus acquisition. Journal of Theoretical Biology 226, 331–340. 14643647 10.1016/j.jtbi.2003.09.011

[CIT0027] Hochholdinger F 2009 The maize root system: morphology, anatomy, and genetics. In: Bennetzen JL Hake SC, eds. Handbook of maize: its biology . New York: Springer, 145–160.

[CIT0028] Hodge A 2004 The plastic plant: root responses to heterogeneous supplies of nutrients. New Phytologist 162, 9–24.

[CIT0029] Hodge A Robinson D Griffiths BS Fitter AH 1999 Why plants bother: root proliferation results in increased nitrogen capture from an organic patch when two grasses compete. Plant, Cell and Environment 22, 811–820.

[CIT0030] Hole DJ Emran AM Fares Y Drew MC 1990 Induction of nitrate transport in maize roots, and kinetics of influx, measured with nitrogen-13. Plant Physiology 93, 642–647. 16667516 10.1104/pp.93.2.642 PMC1062563

[CIT0031] Jenkinson DS 2001 The impact of humans on the nitrogen cycle, with focus on temperate arable agriculture. Plant and Soil 228, 3–15.

[CIT0032] Kell DB 2011 Breeding crop plants with deep roots: their role in sustainable carbon, nutrient and water sequestration. Annals of Botany 108, 407–418. 21813565 10.1093/aob/mcr175 PMC3158691

[CIT0033] Kronzucker HJ Glass ADM Siddiqi MY Kirk GJD 2000 Comparative kinetic analysis of ammonium and nitrate acquisition by tropical lowland rice: implications for rice cultivation and yield potential. New Phytologist 145, 471–476. 10.1046/j.1469-8137.2000.00606.x 33862905

[CIT0034] Kronzucker HJ Siddiqi MY Glass A 1995 Kinetics of NO3 influx in spruce. Plant Physiology 109, 319 – 326. 12228598 10.1104/pp.109.1.319 PMC157591

[CIT0035] Lazof DB Rufty TW Redinbaugh MG 1992 Localization of nitrate absorption and translocation within morphological regions of the corn root. Plant Physiology 100, 1251–1258. 16653113 10.1104/pp.100.3.1251 PMC1075774

[CIT0036] Lozano-Juste J Cutler SR 2014 Plant genome engineering in full bloom. Trends in Plant Science 19, 284–287. 24674878 10.1016/j.tplants.2014.02.014

[CIT0037] Lycklama JC 1963 The absorption of ammonium and nitrate by perennial rye-grass. Acta Botanica Neerlandica 12, 361–423.

[CIT0038] Lynch JP 1995 Root architecture and plant productivity. Plant Physiology 109, 7–13. 12228579 10.1104/pp.109.1.7 PMC157559

[CIT0039] Lynch JP 1998 The role of nutrient-efficient crops in modern agriculture. Journal of Crop Production 1, 241–264.

[CIT0040] Lynch JP Brown KM 2012 New roots for agriculture: exploiting the root phenome. Philosophical Transactions of the Royal Society B: Biological Sciences 367, 1598–1604. 10.1098/rstb.2011.0243 PMC3321693 22527403

[CIT0041] Lynch J Ho MD 2005 Rhizoeconomics: carbon costs of phosphorus acquisition. Plant and Soil 269, 45–56.

[CIT0042] Lynch J Nielsen KL Davis RD Jablokow AG 1997 SimRoot: modelling and visualization of root systems. Plant and Soil 188, 139–151.

[CIT0043] Mahall BE Callaway RM 1992 Root communication mechanisms and intracommunity distributions of two Mojave desert shrubs. Ecology 73, 2145–2151.

[CIT0044] Miller AJ Cramer MD 2004 Root nitrogen acquisition and assimilation. Plant and Soil 274, 1–36.

[CIT0045] Pace GM McClure PR 1986 Comparison of nitrate uptake kinetics parameters across maize inbred lines. Journal of Plant Nutrition 9, 1095–1112.

[CIT0046] Parker JL Newstead S 2014 Molecular basis of nitrate uptake by the plant nitrate transporter NRT1.1. Nature 507, 68–72. 24572366 10.1038/nature13116 PMC3982047

[CIT0047] Postma JA Dathe A Lynch J 2014 *a* The optimal lateral root branching density for maize depends on nitrogen and phosphorus availability. Plant Physiology 166, 590–602. 24850860 10.1104/pp.113.233916 PMC4213091

[CIT0048] Postma J Lynch J 2011 *a* Theoretical evidence for the functional benefit of root cortical aerenchyma in soils with low phosphorus availability. Annals of Botany 107, 829–841. 20971728 10.1093/aob/mcq199 PMC3077978

[CIT0049] Postma JA Lynch JP 2011 *b* Root cortical aerenchyma enhances the growth of maize on soils with suboptimal availability of nitrogen, phosphorus, and potassium. Plant Physiology 156, 1190–1201. 21628631 10.1104/pp.111.175489 PMC3135917

[CIT0050] Postma JA Schurr U Fiorani F 2014 *b* Dynamic root growth and architecture responses to limiting nutrient availability: linking physiological models and experimentation. Biotechnology Advances 32, 53–65. 24012600 10.1016/j.biotechadv.2013.08.019

[CIT0051] Pretty J 2008 Agricultural sustainability: concepts, principles and evidence. Philosophical Transactions of the Royal Society B: Biological Sciences 363, 447–465. 10.1098/rstb.2007.2163 PMC2610163 17652074

[CIT0052] Quaggiotti S Ruperti B Borsa P Destro T Malagoli M 2003 Expression of a putative high-affinity NO_3_ ^–^ transporter and of an H^+^-ATPase in relation to whole plant nitrate transport physiology in two maize genotypes differently responsive to low nitrogen availability. Journal of Experimental Botany 54, 1023–1031. 12598572 10.1093/jxb/erg106

[CIT0053] Quaggiotti S Ruperti B Pizzeghello D Francioso O Tugnoli V Nardi S 2004 Effect of low molecular size humic substances on nitrate uptake and expression of genes involved in nitrate transport in maize (Zea mays L.). Journal of Experimental Botany 55, 803–813. 15020644 10.1093/jxb/erh085

[CIT0054] R Core Team. 2014 R: a language and environment for statistical computing . R Foundation for Statistical Computing, Vienna, Austria, http://www.R-project.org/.

[CIT0055] Rao TP Ito O Matsunaga R Yoneyama T 1997 Kinetics of 15 N-labelled nitrate uptake by maize (Zea mays L.) root segments. Soil Science and Plant Nutrition 43, 491–498.

[CIT0056] Rao KP Rains W 1976 Nitrate absorption by barley. Plant Physiology 57, 55–58. 16659425 10.1104/pp.57.1.55 PMC541962

[CIT0057] Robinson D 1996 Resource capture by localized root proliferation: why do plants bother? Annals of Botany 77, 179–186.

[CIT0058] Robinson D 2001 Root proliferation, nitrate inflow and their carbon costs during nitrogen capture by competing plants in patchy soil. Plant and Soil 232, 41–50.

[CIT0059] Saengwilai P Tian X Lynch JP 2014 Low crown root number enhances nitrogen acquisition from low nitrogen soils in maize (Zea mays L.). Plant Physiology 166, 581–589. 24706553 10.1104/pp.113.232603 PMC4213090

[CIT0060] Serebrovsky AS 1925 ‘Somatic segregation’ in domestic fowl. Journal of Genetics 16, 33–42.

[CIT0061] Shalem O Sanjana NE Hartenian E 2014 Genome-scale CRISPR–Cas9 knockout. Science 343, 84–87. 24336571 10.1126/science.1247005 PMC4089965

[CIT0062] Siddiqi MY Glass ADM Ruth TJ Rufty TW 1990 Studies of the uptake of nitrate in barley. I. Kinetics of 13NO_3_ ^–^ influx. Plant Physiology 93, 1426–1432. 16667635 10.1104/pp.93.4.1426 PMC1062690

[CIT0063] Simunek J Huang K van Genuchten MT 1995 The SWMS_3D code for simulating water flow and solute transport in three-dimensional variably-satured media. Riverside, CA: Salinity Laboratory, US Department of Agriculture.

[CIT0064] Sorgonà A Lupini A Mercati F Di Dio L Sunseri F Abenavoli MR 2011 Nitrate uptake along the maize primary root: an integrated physiological and molecular approach. Plant, Cell and Environment 34, 1127–1140. 10.1111/j.1365-3040.2011.02311.x 21410710

[CIT0065] Sun J Bankston JR Payandeh J Hinds TR Zagotta WN Zheng N 2014 Crystal structure of the plant dual-affinity nitrate transporter NRT1.1. Nature 507, 73–77. 24572362 10.1038/nature13074 PMC3968801

[CIT0066] Sze H Li X Palmgren M 1999 Energization of plant cell membranes by H+-pumping ATPases. Regulation and biosynthesis. The Plant Cell 11, 677–689. 10213786 10.1105/tpc.11.4.677 PMC144215

[CIT0067] Touraine B Glass ADM 1997 NO_3_ ^–^ and CIO_3_ ^–^ fluxes in the chl1-5 mutant of Arabidopsis thaliana. Plant Physiology 114, 137–144. 9159946 10.1104/pp.114.1.137 PMC158287

[CIT0068] Trachsel S Kaeppler SM Brown KM Lynch J 2011 Shovelomics: high throughput phenotyping of maize (Zea mays L.) root architecture in the field. Plant and Soil 341, 75–87.

[CIT0069] Trachsel S Kaeppler SM Brown KM Lynch JP 2013 Maize root growth angles become steeper under low N conditions. Field Crops Research 140, 18–31.

[CIT0070] Trevisan S Borsa P Botton A Varotto S Malagoli M Ruperti B Quaggiotti S 2008 Expression of two maize putative nitrate transporters in response to nitrate and sugar availability. Plant Biology (Stuttgart, Germany) 10, 462–75. 10.1111/j.1438-8677.2008.00041.x 18557906

[CIT0071] Trevisan S Manoli A Ravazzolo L Botton A Pivato M Masi A Quaggiotti S 2015 Nitrate sensing by the maize root apex transition zone: a merged transcriptomic and proteomic survey. Journal of Experimental Botany 66, 3699–3715. 25911739 10.1093/jxb/erv165 PMC4473975

[CIT0072] Tsay Y-F Chiu C-C Tsai C-B Ho C-H Hsu P-K 2007 Nitrate transporters and peptide transporters. FEBS Letters 581, 2290–2300. 17481610 10.1016/j.febslet.2007.04.047

[CIT0073] Veen BW 1980 Energy cost of ion transport. In: Rains DW, ed. Genetic engineering of osmoregulation . Berlin: Springer, 187–198.

[CIT0074] van Vuuren MMI Robinson D Griffiths BS 1996 Nutrient inflow and root proliferation during the exploitation of a temporally and spatially discrete source of nitrogen in soil. Plant and Soil 178, 185–192.

[CIT0075] Waisel Y Eshel A 2002 Functional diversity of various constituents of a single root system. In: Waisel Y Eshel A, eds. Plant roots . New York: Marcel Dekker, Inc, 157–174.

[CIT0076] Wang Y-Y Hsu P-K Tsay Y-F 2012 Uptake, allocation and signaling of nitrate. Trends in Plant Science 17, 458–467. 22658680 10.1016/j.tplants.2012.04.006

[CIT0077] White JW Rassweiler A Samhouri JF Stier AC White C 2014 Ecologists should not use statistical significance tests to interpret simulation model results. Oikos 123, 385–388.

[CIT0078] Wirth J Chopin F Santoni V Viennois G Tillard P Krapp A Lejay L Daniel-Vedele F Gojon A 2007 Regulation of root nitrate uptake at the NRT2.1 protein level in Arabidopsis thaliana. Journal of Biological Chemistry 282, 23541–23552. 17573350 10.1074/jbc.M700901200

[CIT0079] World Bank. 2014 Health nutrition and population statistics: population estimates and projections.

[CIT0080] Wullschleger SD Lynch JP Berntson GM 1994 Modeling the belowground response of plants and soil biota to edaphic and climatic change. What can we expect to gain? Plant and Soil 165, 149–160.

[CIT0081] York LM Lynch JP 2015 Intensive field phenotyping of maize (Zea mays L.) root crowns identifies phenes and phene integration associated with plant growth and nitrogen acquisition. Journal of Experimental Botany 66, 5493–5505. 26041317 10.1093/jxb/erv241 PMC4585417

[CIT0082] York LM Nord EA Lynch JP 2013 Integration of root phenes for soil resource acquisition. Frontiers in Plant Science 4, 1–15. 24062755 10.3389/fpls.2013.00355 PMC3771073

[CIT0083] Zhan A Lynch JP 2015 Reduced frequency of lateral root branching improves N capture from low N soils in maize. Journal of Experimental Botany 66, 2055–2065. 25680794 10.1093/jxb/erv007 PMC4378636

[CIT0084] Zhan A Schneider H Lynch JP 2015 Reduced lateral root branching density improves drought tolerance in maize. Plant Physiology 168, 1603–1615. 26077764 10.1104/pp.15.00187 PMC4528736

[CIT0085] Zhang C Postma JA York LM Lynch JP 2014 Root foraging elicits niche complementarity-dependent yield advantage in the ancient ‘three sisters’ (maize/bean/squash) polyculture. Annals of Botany 114, 1719–1733. 25274551 10.1093/aob/mcu191 PMC4416130

[CIT0086] Zhang D 1999 Donald’s ideotype and growth redundancy: a game theoretical analysis. Field Crops Research 61, 179–187.

[CIT0087] Zhu J Kaeppler SM Lynch JP 2005 Topsoil foraging and phosphorus acquisition efficiency in maize (Zea mays). Functional Plant Biology 32, 749–762. 10.1071/FP05005 32689172

[CIT0088] Zhu J Lynch JP 2004 The contribution of lateral rooting to phosphorus acquisition efficiency in maize (Zea mays) seedlings. Functional Plant Biology 31, 949–958. 10.1071/FP04046 32688963

